# Ectopic Cushing’s syndrome. A metastatic hepatic neuroendocrine tumour: case report and literature review

**DOI:** 10.1093/jscr/rjag848

**Published:** 2026-09-21

**Authors:** Mazin Ahmed Subhy Alsheikhly, Shafik Samir Mansour, Hussam Telfah, Ahmed Elaffandi

**Affiliations:** Hepatobiliary and Liver Transplant Surgery, Hamad Medical Corporation, Doha, Qatar; Endocrinology, Hamad Medical Corporation, Doha, Qatar; Department of Laboratory Medicine and Pathology, Hamad Medical Corporation, Doha, Qatar; Hepatobiliary and Liver Transplant Surgery, Hamad Medical Corporation, Doha, Qatar

**Keywords:** hepatic neuroendocrine tumour, ectopic Cushing's syndrome, hepatectomy, DOTATATE, hypercortisolism

## Abstract

This report describes a rare instance of an adrenocorticotropic hormone (ACTH)-secreting metastatic hepatic neuroendocrine tumour (MHNET) exhibiting as an ectopic Cushing’s syndrome. A 41-year-old woman with a history of supraglottic neuroendocrine tumour presented with rapid weight gain, severe hypokalemia, and hypercortisolism. Pituitary imaging was unremarkable. However, the DOTATATE PET/CT showed a large liver lesion. Initiating etomidate infusion caused a rapid drop in cortisol, leading to severe vasoplegic and septic shock, which delayed the surgery. The patient required intensive care stabilization pre-operatively and treatment with osilodrostat, which was then followed by surgical management: left lateral hepatectomy and gastric wedge resection. ACTH levels markedly decreased postoperatively, reflecting successful management. This emphasizes that careful medical management and multidisciplinary collaboration are essential in treating severe, potentially life-threatening ectopic hypercortisolism.

## Introduction

We discuss a rare case of a neuroendocrine tumour developing as a functional metastatic hepatic neuroendocrine tumour (MHNET) that presented as ectopic Cushing’s syndrome. Neuroendocrine tumours usually originate from either the tubular gastrointestinal tract or the pancreas, which account for ~50% of all neuroendocrine tumours, or from the bronchopulmonary tree, with an incidence of ~30% [1]. Since 1958, ~300 cases of hepatic neuroendocrine tumours (HNET) have been reported [2]. While most are non-functional neuroendocrine tumours, our literature review noted that only a few cases reported since 2000 have presented as ectopic adrenocorticotropic hormone-secreting tumours [3]. This rarity may make the diagnosis, management, and surgical approaches challenging. Collaborative efforts between the endocrine team and hepatobiliary surgical experts were necessary to accurately diagnose and manage the patient in our discussion.

## Case presentation

A 41-year-old woman with a history of excised neuroendocrine tumour in the left supraglottic area presented with rapid weight gain (~15 kg over months), facial and palm hyperpigmentation, fatigue, and muscle weakness. Despite correcting hypokalemia, potassium remained low at 3 mmol/L. Dexamethasone suppression test showed elevated adrenocorticotrophic hormone (ACTH) (139 pg/ml) and non-suppressed cortisol (1327 nmol/L), indicating Cushing's syndrome. Elevated midnight salivary cortisol was confirmed twice.

**Figure 1:**
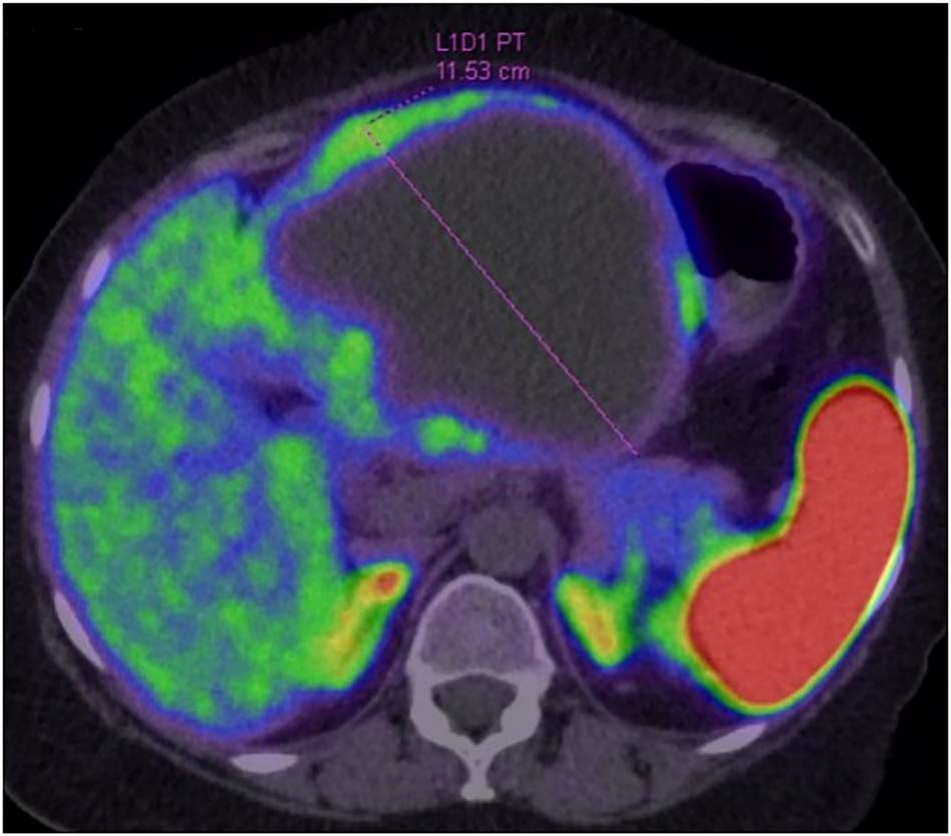
PET DOTATATE scan showing hepatic lesion with moderate to high DOTA uptake.

**Figure 2:**
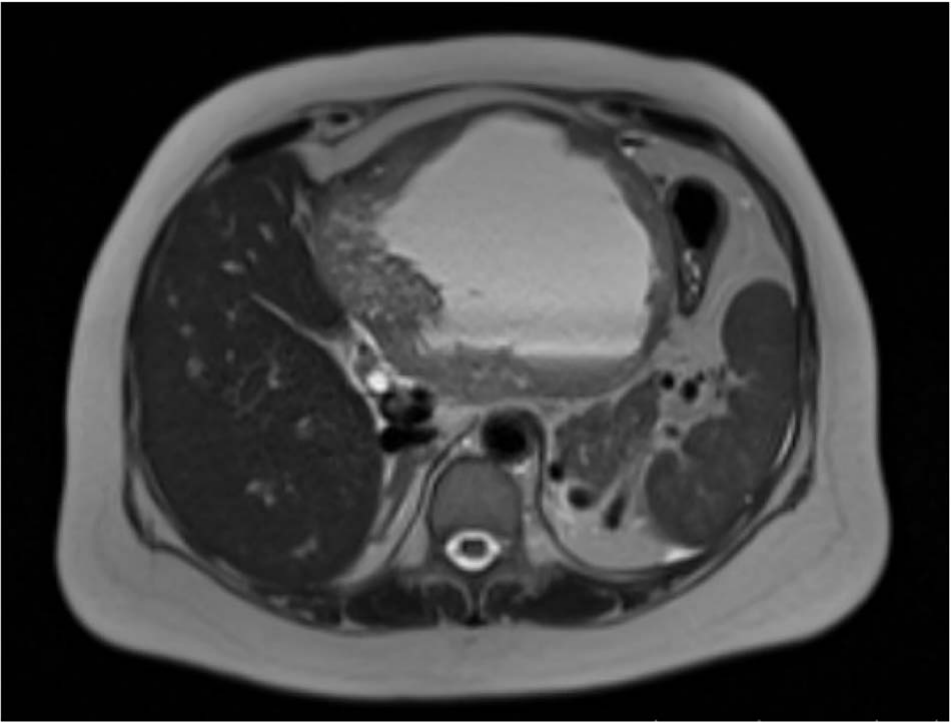
MRI scan showing a thick-walled cystic lesion [14.0 × 12.7 × 9.8 cm] inseparable from the left lobe of the liver.

Imaging with neck and thorax computed tomography (CT) showed no recurrence. Pituitary magnetic resonance imaging (MRI) was normal, with no adenoma. Whole-body PET DOTATATE revealed a large abdominal lesion (Fig. 1), and MRI abdomen showed the lesion to be hepatic in origin (Fig. 2).

Endocrinology started osilodrostat 2 mg, and surgery was considered resectable after multidisciplinary assessment, pending patient optimization. Due to worsening mental status and poor control despite increasing medication, the patient was transferred to Intensive Care Unit (ICU) for etomidate infusion at 0.05 mg/kg/hr. Rapid cortisol suppression caused vasoplegic and septic shock, disseminated intravascular coagulation (DIC), and multi-organ failure, delaying surgery and extending ICU stay.

Importantly, at the time of hemodynamic collapse, serum cortisol dropped to 800 nmol/L, which remains well above levels associated with adrenal insufficiency. However, the sudden decline, along with potential underlying sepsis and the patient’s critical haemodynamics, may have triggered a physiological withdrawal response. This response manifested as DIC, septic shock, and multi-organ failure, rather than a true classical adrenal crisis being the primary cause of shock. It suggests unmasking of critical illness physiology that was previously hidden by extreme conditions of hypercortisolism.

Further ICU optimization was performed; the patient needed triple vasopressors and mechanical ventilation. After a prolonged stay and meticulous care, the patient recovered from multi-organ failure, sepsis was controlled, and a feasible surgical window became available.

Discussions in hepatobiliary multidisciplinary team considered IR embolization or partial ablation to reduce the lesion and enable resection. However, the patient's condition and family hesitations favored surgical resection. Risks and complications were discussed with the family in a multidisciplinary team meeting.

Due to high surgical risk amid critical illness, it was decided to stabilize biochemically with osilodrostat and delay surgery until improvement. Osilodrostat increased to 3 mg twice daily, stabilizing cortisol levels. With clinical improvement, the patient underwent open left lateral hepatectomy and gastric wedge resection. Intraoperatively, a large hepatic, partially cystic mass adherent to the stomach was identified and completely resected. An estimated blood loss of 200 ml, and 1 unit of blood transfused. The operation lasted ~420 minutes.

Perioperative optimization to minimize abrupt adrenal insufficiency was achieved by administering a stress dose of 100 mg IV hydrocortisone at anesthesia induction, followed by an infusion of 200 mg/24 h for 3 days, which was then reduced to 100 mg/24 hr. Postoperative ACTH dropped to 3.3 pg/ml, an excellent response to surgical excision. The patient was maintained on steroids postoperatively and the dose was adjusted daily by the endocrine team.

**Figure 3:**
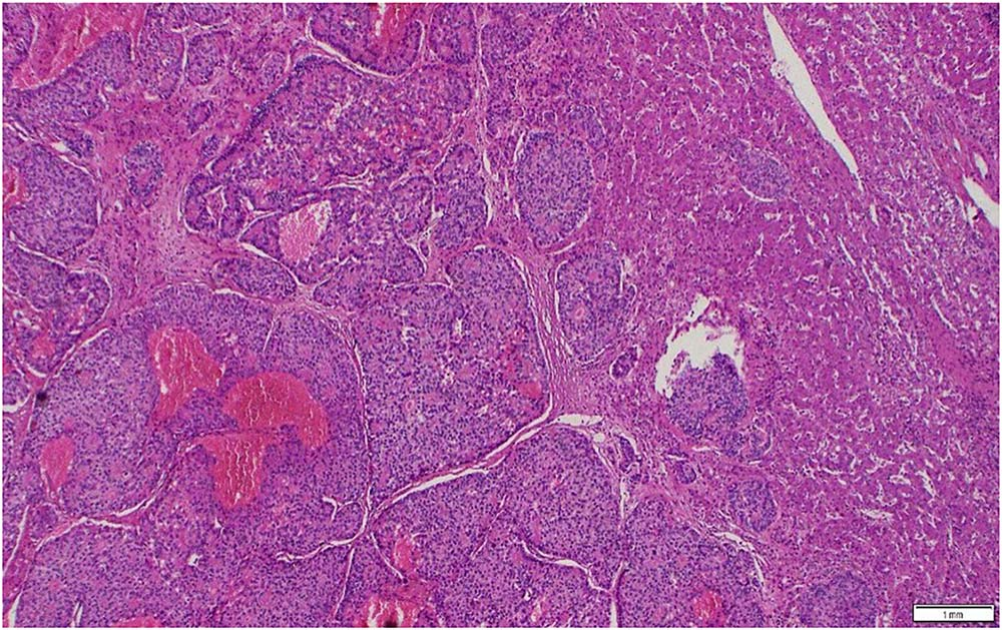
Low-power view of the tumor with adjacent liver tissue. H&E ×2.

**Figure 4:**
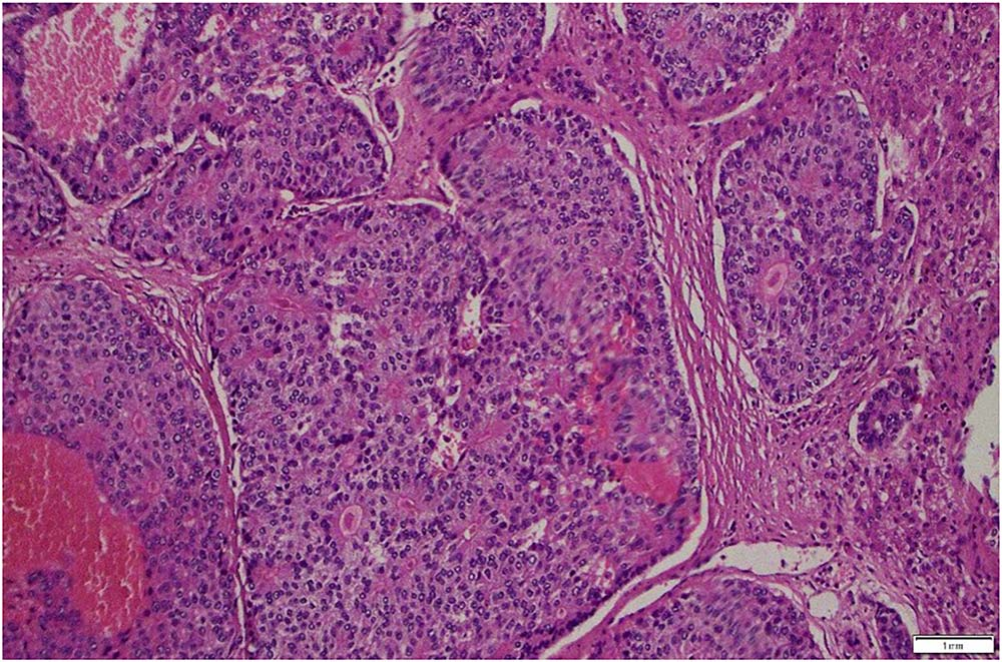
Islands of tumor, including pseudo-rosettes. H&E ×5.

**Figure 5:**
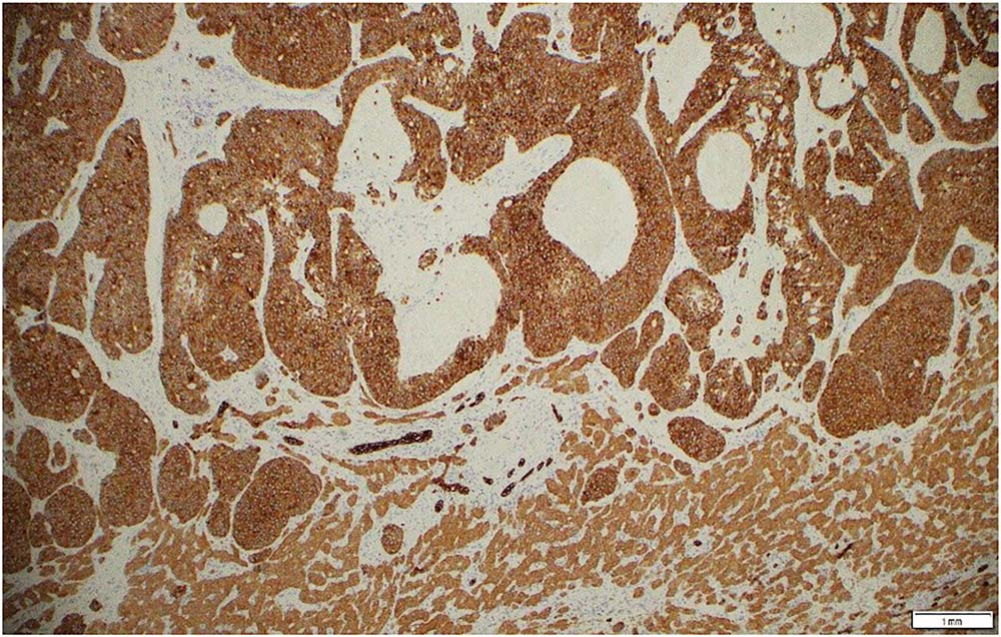
Diffuse positivity with CKAE1/3.

**Figure 6:**
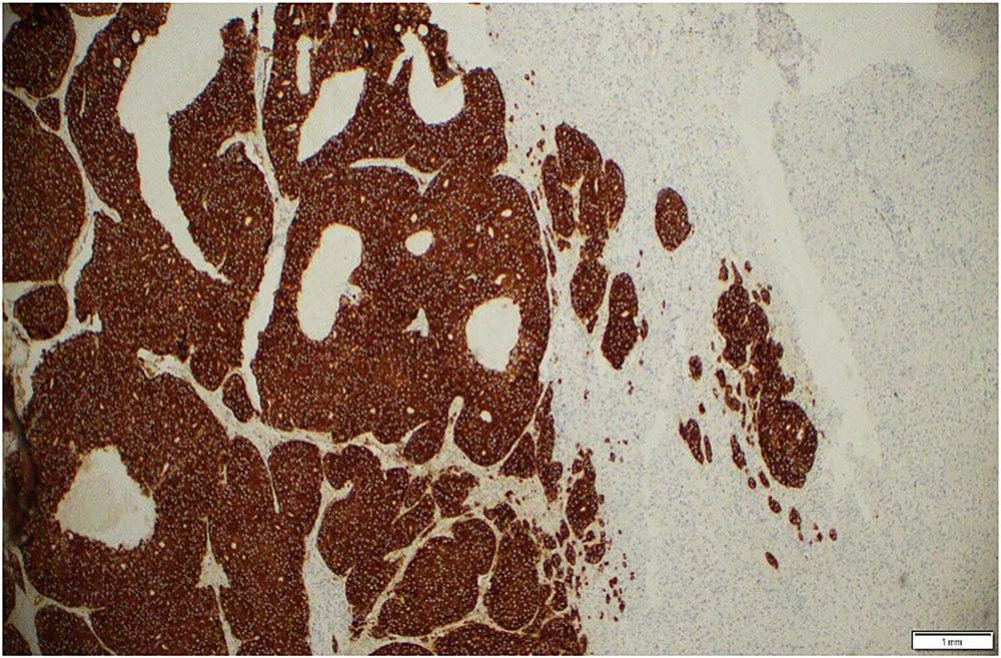
Diffuse positivity with synaptophysin.

**Figure 7:**
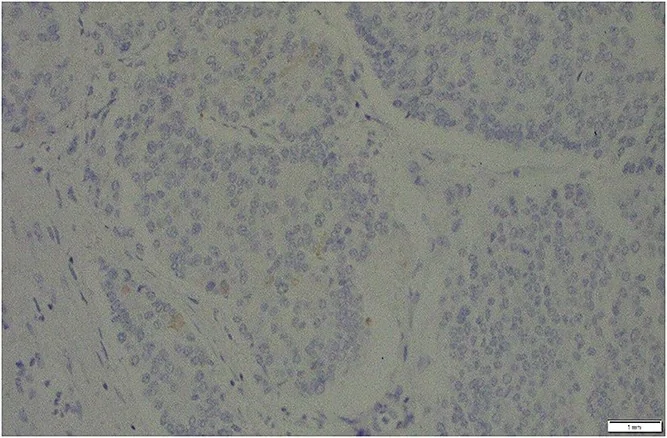
Occasional cells stain faintly with ACTH (cell marque antibodies, rabbit polyclonal antibody 0.6 μg/ml).

Final pathology (Figs 3–7) showed a grade 2 metastatic well-differentiated neuroendocrine tumour with an R0 resection margin. Histopathological analysis revealed multiple sections of an epithelioid tumour arranged in nests, islands, and trabeculae of epithelioid cells with amphophilic cytoplasm and focal eosinophilic secretion. Pseudo-rosettes were present in some areas. Immunostaining confirmed the diagnosis of a clinically suspected well-differentiated neuroendocrine neoplasm secreting ACTH.

After months of ICU, Endocrine, and Hepatobiliary care, the patient was transferred to Rehab, regaining function, maintaining daily needs, and ambulating with partial support. Her hypothalamic–pituitary–adrenal axis remains suppressed, requiring daily hydrocortisone until recovery.

Recovery was complicated by an acute abdomen and small bowel perforation, necessitating emergency surgery. A segment of ileum was resected, and a double-barrel ileostomy was created. Pathology showed inflammation and ulceration, with no recurrent disease. Bowel anastomosis was deferred due to chronic hydrocortisone use.

Follow-up MRI and DOTATATE scan at 6 months showed nodular lesions at the hepatic resection margin, identified as postoperative inflammatory changes and cholangitis, with no recurrent disease. The patient will continue follow-up with a new scan and endocrine management, including hydrocortisone 10 mg daily and close monitoring for adrenal insufficiency.

## Discussion

ACTH-secreting hepatic neuroendocrine tumours are among the rarest causes of ectopic Cushing’s. HNETs make up <2% of all neuroendocrine neoplasms and are characterized by their ability to secrete hormones [3, 4]. ACTH secretion by HNETs is even rarer, with only a few cases reported since 2000 [3]. These tumours can develop at any age, from children to the elderly, and can affect both sexes [2, 5].

Ectopic ACTH causes paraneoplastic Cushing’s, presenting with rapid symptoms like moon facies, obesity, myopathy, diabetes, resistant hypertension, hypokalemia, and psychiatric features [6]. Severe metabolic issues such as extreme hypertension, new-onset diabetes, and refractory hypokalemia stem from cortisol's mineralocorticoid effects [4].

Cushing’s syndrome involves excess corticotropin (ACTH), called ACTH-dependent Cushing syndrome, making up ~80% of cases. This includes pituitary hypersecretion of ACTH (70%), ectopic ACTH from nonpituitary tumours (10%), and ectopic corticotropin-releasing hormone from nonhypothalamic tumours causing pituitary hypersecretion (10%). ACTH-independent Cushing syndrome, ~20%, involves autonomous adrenal cortisol secretion [7].

The challenge in treating Cushing’s syndrome is determining if it's ACTH-dependent. After suspicion, abnormal cortisol can be accessed via urine-free cortisol (UFC) or late-night salivary cortisol (LNSC) [8]. LNSC has 97% sensitivity and 97.5% specificity; it is easy for patients to perform with some contamination prevention restriction [8]. Alternatively, UFC can be used; however, it can be cumbersome for the patient, and it falls behind LNSC in terms of sensitivity and specificity [8].

ACTH dependency can be identified by measuring plasma ACTH levels; normal or high ACTH levels indicate ACTH-dependent Cushing’s syndrome, and further imaging with MRI of the pituitary gland is necessary to check for an adenoma [9]. In Cushing’s disease (pituitary adenoma-dependent ACTH), glucocorticoid receptors can still suppress ACTH. A high-dose dexamethasone suppression test and imaging help distinguish ectopic from non-ectopic sources. Ectopic ACTH sources lack receptors and do not suppress ACTH and cortisol [10].

In our case, after the establishment of hypercortisolism and failure of ACTH suppression, a negative pituitary MRI result raised the question of the ectopic source. ^68^Ga-DOTATATE scan was the key to diverting the focus towards the hepatic lesion. ^68^Ga-DOTATATE is a modified (Tyr3)-octreotide molecule covalently linked to 1,4,7,10-tetra-azacyclododecane-1,4,7,10-tetra-acetic acid (DOTA) combined with the radioactive ^68^Ga isotope [8], with the ability to localize to ~65% of ectopic ACTH-secreting NETs [11].

The primary approach to treating ectopic ACTH-secreting tumours is surgical resection; however, this must be complemented by suitable medical therapy to manage hypercortisolism, particularly when the tumour is unresectable, occult, or metastatic. Medical management includes adrenal steroidogenesis inhibitors [12], which rapidly decreases cortisol synthesis. The most frequently employed agents are ketoconazole, metyrapone, and etomidate, with the latter administered intravenously in critically ill patients [13]. Additionally, mitotane and the newer agent, osilodrostat, are effective options. Combination therapy utilizing these agents may be employed in severe or refractory cases to attain adequate cortisol control [12].

**Figure 8:**
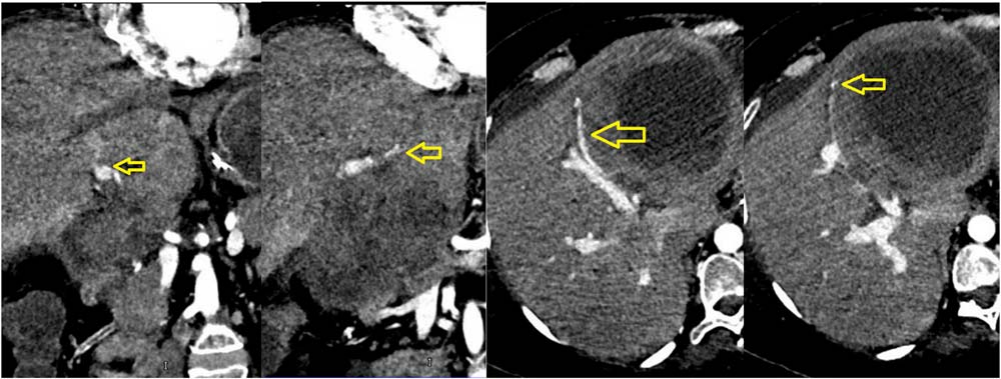
Abdominal CT angiography showing the left hepatic artery supplying the lesion.

Other adjunct therapies for surgical resection include embolization. During multidisciplinary team discussions, IR was suggested to address the lesion, even partially, to control hypercortisolism and reduce tumour burden. Abdominal CT angiography (Fig. 8) shows the left hepatic artery supplying the lesion. Transarterial embolization effectively treats MHNET with hormonal symptoms or growth, as these tumours are highly vascularized, mainly supplied by the hepatic artery, while healthy liver tissue is supplied via the portal vein. Intra-arterial therapies include TAE, TACE, and Y-90 radioembolization, successfully controlling tumours and symptoms [14].

Perioperative optimization aims to reduce the risks associated with tumour manipulation and hormonal surgery. Since the main perioperative risk is sudden adrenal insufficiency, a standardized protocol was used. This involves administering a stress dose of 100 mg IV hydrocortisone during anesthesia induction, followed by a continuous infusion of 200 mg over 24 h [15]. This approach provides adequate cortisol coverage during surgical stress and helps maintain haemodynamic stability.

The literature review (Table 1) focuses on MHNET associated with Cushing's syndrome. These rare, aggressive tumours primarily arise from gastroenteropancreatic sources, such as rectal, pancreatic, or small-bowel NETs, and liver metastases can drive ectopic ACTH production. Notable cases, such as that of Sandozi *et al.* [3], demonstrate rapid deterioration with symptoms including anasarca, hypokalemia, and hyperglycemia due to diffuse bilobar liver NETs with bone spread, often resulting in death despite supportive treatment, highlighting their late-stage presentation. Most cases, including Sandozi's, have poor outcomes, with death usually caused by metabolic crisis or disease progression. In contrast, well-differentiated liver-dominant tumours, such as those described by Huang *et al.* [22], can achieve partial stabilization. Overall, mortality across reported cases is high and tends to occur early, driven by tumour progression, sepsis, cardiovascular events, and complications of hypercortisolism. This case emphasizes the importance of meticulous planning and multidisciplinary collaboration to optimize patient outcomes.

**Table 1:** Table summary of studies about MHNET associated with Cushing's syndrome.

Author, Year	Age, Sex	Primary / Metastatic	Diagnosis	Major features	Treatment	Outcome
Shah *et al.* 2007 [16]	65 M	Primary	Biochemical diagnosis and liver biopsy	Classic Cushing syndrome in PHNET	Surgical resection of hepatic lesions	Symptomatic and biochemical improvements
Mehta *et al.* 2020 [17]	49 F	Metastatic	Biochemical diagnosis + imaging + biopsy	ACTH, pancreatic NET; Cushing’s syndrome	Distal pancreatectomy with liver metastasectomy and endocrine therapy	Remission of Cushing’s
Cipriani *et al.* 2022 [18]	74 F	Metastatic	Biochemical diagnosis + imaging + biopsy	Cushing for hepatic metastases	Medical therapy + systemic oncologic treatment	Partial hormonal control
Mineur *et al.* 2021 [19]	50 M	Metastatic	Biochemical diagnosis and liver biopsy	Cushing’s GI NET with hepatic spread	Cortisol-lowering drugs + systemic NET therapy	Transient improvement
Waghela *et al.* 2022 [6]	63 F	Primary	Biochemical diagnosis and histopathological confirmation	Ectopic ACTH, Cushing’s; unresectable PHNET	Liver-directed Transarterial radioembolization and medical endocrine management	Significant biochemical and clinical response; ongoing disease but controlled hypercortisolism.
Zheng *et al.* 2022 [20]	60 F	Metastatic	Biochemical diagnosis + imaging + biopsy	Pancreatic NET with liver metastases, Cushing’s syndrome	Tumour-directed surgical resection, systemic NET therapy, and chemotherapy	Improvement in Cushing’s syndrome: Poor prognosis
Sandozi *et al.* 2023 [3]	71 F	Metastatic	Biochemical diagnosis and biopsy	Paraneoplastic Cushing	Supportive endocrine control and oncological therapy	Fatal outcome
Zhang *et al.* 2023 [21]	65 M	Metastatic	Biochemical diagnosis and biopsy	Cushing’s disease: ACTH-producing NEC of the gallbladder with liver metastases	Attempted systemic therapy, however, stopped due to rapid deterioration; Supportive Care.	Fatal outcome
Huang *et al.* 2025 [22]	46 F	Metastatic	Biochemical diagnosis + imaging + biopsy	Cushing**’**s disease: Pancreatic NET with metastatic spread, including the liver.	Medical cortisol control + Systemic NET therapy + surgical resection	Partial hormonal Responses
